# Establishment of a large semi-field system for experimental study of African malaria vector ecology and control in Tanzania

**DOI:** 10.1186/1475-2875-7-158

**Published:** 2008-08-20

**Authors:** Heather M Ferguson, Kija R Ng'habi, Thomas Walder, Demetrius Kadungula, Sarah J Moore, Issa Lyimo, Tanya L Russell, Honorathy Urassa, Hassan Mshinda, Gerry F Killeen, Bart GJ Knols

**Affiliations:** 1 Division of Infection and Immunity, University of Glasgow, G12 8TA, Glasgow, UK; 2 Public Health Entomology Unit, Ifakara Health Institute, Ifakara, P.O Box 53, Tanzania; 3 Maintenance Unit, Tanzanian Training Centre for International Health, Ifakara, P.O. Box 39, Tanzania; 4 School of Biology Sciences, University of Durham, DH1 3LE, Durham, UK; 5 Laboratory of Entomology, Wageningen University and Research Centre, P.O. Box 8031, 6700 EH, Wageningen, The Netherlands

## Abstract

**Background:**

Medical entomologists increasingly recognize that the ability to make inferences between laboratory experiments of vector biology and epidemiological trends observed in the field is hindered by a conceptual and methodological gap occurring between these approaches which prevents hypothesis-driven empirical research from being conducted on relatively large and environmentally realistic scales. The development of Semi-Field Systems (SFS) has been proposed as the best mechanism for bridging this gap. Semi-field systems are defined as enclosed environments, ideally situated within the natural ecosystem of a target disease vector and exposed to ambient environmental conditions, in which all features necessary for its life cycle completion are present. Although the value of SFS as a research tool for malaria vector biology is gaining recognition, only a few such facilities exist worldwide and are relatively small in size (< 100 m^2^).

**Methods:**

The establishment of a 625 m^2 ^state-of-the-art SFS for large-scale experimentation on anopheline mosquito ecology and control within a rural area of southern Tanzania, where malaria transmission intensities are amongst the highest ever recorded, is described.

**Results:**

A greenhouse frame with walls of mosquito netting and a polyethylene roof was mounted on a raised concrete platform at the Ifakara Health Institute. The interior of the SFS was divided into four separate work areas that have been set up for a variety of research activities including mass-rearing for African malaria vectors under natural conditions, high throughput evaluation of novel mosquito control and trapping techniques, short-term assays of host-seeking behaviour and olfaction, and longer-term experimental investigation of anopheline population dynamics and gene flow within a contained environment that simulates a local village domestic setting.

**Conclusion:**

The SFS at Ifakara was completed and ready for use in under two years. Preliminary observations indicate that realistic and repeatable observations of anopheline behaviour are obtainable within the SFS, and that habitat and climatic features representative of field conditions can be simulated within it. As work begins in the SFS in Ifakara and others around the world, the major opportunities and challenges to the successful application of this tool for malaria vector research and control are discussed.

## Background

Recent advances in genomics and bioinformatics are allowing science to test the boundaries of reductionism as never before: how well can biological processes observed at the level of individual molecules, genes or cells predict the behaviour of more complex systems such as whole organs, individuals, or populations? These exciting technological developments have generated renewed interest within existing, more long-established biological disciplines to seek out empirical tools for quantifying and testing the relationship between phenomena occurring at different levels of biological organization in order to generate better predictions. One such field is medical entomology. Typically research in medical entomology is conducted at two very different scales: the first being laboratory-based studies of arthropod disease vectors under controlled insectary conditions, and the second being large-scale epidemiological surveys of their abundance and distribution in nature. While the former approach is clearly advantageous for identifying potential biological mechanisms, and the latter for generating hypotheses from correlations, it is increasingly recognized that the ability to make inferences across these scales is hindered by a conceptual and methodological gap in between that limits our ability to conduct hypothesis-driven empirical research on relatively large and environmentally realistic scales [1].

Arguably for the first time in the history of their discipline, medical entomologists now find themselves in the unique position of having both the need for such experimental initiatives recognized [2], and the financial support to create them becoming available through new funding streams in global health. The need to fill the research gap between laboratory and field is also stimulated by awareness that although current approaches to important vector-borne diseases such as malaria based on artemisinin-based combination therapies and insecticide treated bed nets are proving successful [3-7], their long-term effectiveness may be undermined by the emergence of drug and insecticide resistance [8-10]. Consequently, the need for new strategies that exploit novel aspects of vector genetics, physiology, behaviour and ecology are increasingly needed. These innovations must be drawn from an understanding of vector biology within natural transmission settings if they are to yield rapid, locally appropriate strategies for disease control.

While almost all new approaches to vector control could benefit from a closer integration of laboratory and field perspectives [11], the most prominent candidate is the development of transgenic parasite resistant and/or sterile vectors whose release into the wild could reduce disease transmission by reducing parasite and/or vector populations [12-15]. In the past decade, the genetic transformation of a number of important disease-transmitting mosquito species has become possible [16-20]. Transgenes have been identified that deliver effector molecules that substantially reduce the development of rodent malaria parasites [21-23] and human dengue virus within mosquitoes [24], which has fuelled optimism that mass-release of laboratory-reared genetically-modified individuals could reduce disease transmission. The greatest unknown with respect to the feasibility of this approach is whether genetically-modified mosquitoes would be able to survive and successfully compete for mates against their wild counterparts outside of the confines of the laboratory. Initial laboratory studies indicated that transgenes impose fitness costs which reduce the reproductive success of the mosquito bearer [25-27]. A recent study suggests this disadvantage can be reduced by use of out-crossed mosquito lines [28], although so far only under conditions where exposure to parasites is substantially greater than mosquitoes encounter in the wild. While this improvement is encouraging, it does not address the problem that all laboratory-reared mosquitoes, regardless of their genotype, may have poor competitive ability in the wild. For example, recent comparative analysis of *Anopheles gambiae s.s*. in captivity and in nature in southern Tanzania suggest free-living males are larger and have greater lipid reserves than those reared under apparently optimal laboratory conditions [29]. Regardless of whether this reduction in energetic reserves was due to selection for smaller individuals during the colonization process and/or sub-optimal conditions of insectary environments, it suggests laboratory-reared mosquitoes could be at a sizeable disadvantage to their wild counterparts.

Additional studies have shown that the mating success of male mosquitoes depends on subtle variation in environmental conditions experienced during larval development [30,31], which may not be fully captured in mass-rearing facilities. These limitations are thought to have been responsible for the failure of many genetic control trials during the 1970's and 80's, which found that laboratory-reared male mosquitoes were unable to compete in the wild [32]. Clearly, to avoid repeating these failures with the new generation of transgenic mosquitoes, intermediary testing grounds between the laboratory and field within disease-endemic countries are needed.

Semi-Field Systems (SFS) have been proposed as the best mechanism for bridging this gap. A semi-field system is here defined as an enclosed environment, ideally situated within the natural ecosystem of the target disease vector and exposed to ambient environmental conditions, within which all features necessary for its lifecycle completion are present [33]. In the case of mosquito vectors of human disease, this typically involves a large outdoor cage in which the movement of the disease vector of interest either in or out of the unit is restricted by netting, and within which features such as aquatic larval habitats, blood hosts for adult females, sugar sources (plants) for adults, appropriate resting sites (houses, cattle sheds, etc.) and environmental features (e.g swarm markers to stimulate mating), are present. There are no general guidelines for the appropriate size of such a unit, but ideally it should be large enough to sustain a population of similar density to that encountered in the target environment for numerous generations.

This definition of a SFS differs from others that apply 'semi-field' to studies that actually involve observation of vectors in a non-contained setting or habitat, where only one part of its life cycle is present [34]. A major goal of SFS is to establish multiple generations of a vector population within a contained setting, without outside intervention [35] in addition to facilitating short-term behavioural or ecological studies based on a single cohort. The main advantage of this approach is that because the abundance and composition of vectors within the SFS can be known, and if desired experimentally manipulated (either at the time of introduction, or through removal of some target individuals), much more precise estimates of the value and variability of demographic and life-history parameters can be obtained than would be from the field. Additionally, they allow researchers to conduct high throughput assays of control tools and ecological phenomena year round without risk of exposure to infection, as all mosquitoes used within the SFS will be free of parasites.

The concept of simulating the natural environment within contained settings in order to experimentally test ecological hypotheses does not originate in medical entomology. This approach has a long history in aquatic ecology, where hundreds of studies have successfully employed pond meso- and microcosms to examine the impact of biotic and abiotic factors on population and community dynamics [36]. Furthermore, neither is this approach new within medical entomology. Almost 70 years ago, Hackett and Bates [37] commented on this need for ecological experimentation within natural disease transmission settings: "The study of behavior under natural, semi-natural and laboratory conditions necessitates locating the laboratory at the source of material. Self evident as this may seem, there are very few laboratories of this kind functioning at present in malarial regions". Since that time, only a handful of attempts have been made to create large-scale research facilities within semi-natural conditions in disease endemic settings, with the majority being initiated only in the last decade (Table 1). Early work in Albania and India used outdoor cages (< 75 m^2^) to conduct basic ecological observation of anopheline species [37,38]. Thirty years later this approach was revived for comparative evaluation of different genetically-based population suppression methods for the Indian vectors *Aedes aegypti *and *Culex fatigans *[35,39,40] but was discontinued after the abandonment of the Sterile Male Release programme that motivated this research [41]. Within the last decade, several research programmes in Africa, Asia, Europe and Australia have revitalized SFS for examination of mosquito vector ecology and control (Table 1). This approach has been used particularly productively in western Kenya [33], where SFS studies of the malaria vector *An. gambiae s.s. *within 85 m^2 ^modified greenhouses have yielded valuable insights into basic ecology and vector-parasite interactions [42-44] and novel control and monitoring methods [45-48]. Here the establishment of what is currently the largest SFS in the world for the purpose of experimental study of the ecology and control of African anopheline malaria vectors is described. This facility was built over a two-year period at the Ifakara Health Institute (2004–2006) and is the site of several new studies on vector behaviour, ecology and control.

**Table 1:** Previous and current location, size, target species and research aims of Semi-Field Systems (SFS) established for mosquito vector research.

Country	Year*	Dimensions (m)	Number of units	Mosquito Species	Purpose	Refs
Albania	1939	10 × 5 × 6	1	Various European anophelines	Basic ecological studies	[37]
India – Madras	1942	12.2 6.1 × 3.05	3	*An. culifacies*	Basic ecological studies, evaluation of genetic control strategies for population suppression	[38]
India – Delhi	1976	5.6 × 3.3 × 2.1	1	*Ae. aegypti Cx. Fatigans*		[35,39,40]
Kenya	2002	11.4 × 7.1 × 4.4	7	*An. gambiae s.s.*	Basic ecological studies, vector-malaria parasite interactions, evaluation of novel trap designs and repellents	[33,42-48]
Thailand	2003	10 × 10 × 4	1	*Ae. aegypti*	Basic ecological studies	[90]
Tanzania – Muheza	2003	12.2 × 8.2 × 4.6	3	*An. gambiae s.s Cx. quinquefasciatus*	Evaluation of trapping methods, training and basic ecological studies	No publ.
Sudan	2006	18 × 8 × 2.75	3	*An. arabiensis*	Fitness of sterilized males, basic ecological studies	[65]
Tanzania – Ifakara	2007	29.8 × 21 × 7.1	4	*An. gambiae s.s An. arabiensis*	Basic ecological studies, evaluation of trapping methods and repellents	This paper
Australia	2008	17 × 9 × 4.3	2	*Ae. aegypti*	Assessment of biocontrol strategy using Wolbachia, basic ecological studies	No publ.
Austria	TBC	25 × 10 × 3	TBD	*An. arabiensis*	Research on Sterile Insect Technique	No publ.

Year refers to the time when the first research publication from these facilities was published, or year of establishment in cases where no published references to these facilities are yet available ('TBC' = to be constructed).

## Materials and methods

### Study site

The SFS was estabished at the Ifakara Health Institute (IHI) located in the Kilombero district of southern Tanzania. Malaria transmission intensities within this area are amongst the highest described for sub-saharan Africa [49,50]; with annual entomological inoculation rates exceeding three hundred infectious bites a year in some locations [49,51,52]. The major malaria vectors in this region are *Anopheles arabiensis, An. gambiae s.s*. and *An. funestus *[52-54].

### SFS site selection

The crucial first step in establishing a SFS is identifying an appropriate site that adequately captures the environmental conditions experienced by local mosquito species. Additional logistic criteria include ease of access by research personnel and electricity/water supply, being situated where potential hazards to surrounding residents arising from accidental vector release are negligible, and continual monitoring by security staff is possible. Trade-offs may arise in attempting to maximize all these criteria at particular locations which will require careful case-by-case consideration. For example, it has been suggested that the best way to limit hazards posed by unintentional release of mosquitoes into the environment would be to build containment units as far away from communities as possible [55]. However, the majority of SFS currently in existence and being planned are located within disease-endemic settings in the developing world. In many of these settings, access to roads, water, an electrical supply, and reliable 24-hour surveillance is possible only near towns or cities. In balancing these components of potential risk, it was decided to select a site for the SFS that is within the campus of the IHI, which is located in Ifakara town. By building within the fenced-off perimeter of the research centre, it was possible to ensure constant surveillance and containment, and strictly control those who had access to the SFS.

Another key factor in the site selection process for SFS is the availability of background data on the dynamics of local vector populations and their disease transmission ability [55]. This information is essential to examine how closely the behaviour, life-history and population dynamics of contained vectors represent those of the wild. As mosquitoes in the SFS will be exposed to many of the same environmental conditions as those of neighbouring populations (e.g temperature, humidity, vegetation), it is anticipated they will be subject to similar selective forces. However, one deviation from complete 'naturalness' was made in the IHI SFS by covering its roof with polyethylene plastic; a decision taken on the basis that this compromise would permit experimental manipulation of rainfall in future experiments. How this modification influences the environmental suitability of the SFS relative to ambient conditions can be assessed by comparison of mosquito population dynamics in the SFS with those of the surrounding area. An advantage of selecting a site in Ifakara was that substantial baseline epidemiological and entomological information on the dynamics of malaria and *Anopheles *populations in the area is already available [50,54,56,57]. Additionally, detailed knowledge of mosquito ecology exists for the Kilombero valley, and new studies specifically addressing the mating biology [29-31,58] and population genetics (Ng'habi et al., in prep.) of *An. gambiae *and *An. arabiensis *within this region were initiated concurrently with the establishment of the SFS.

### Planning and design

Given that Ifakara town is occasionally subject to flooding during the rainy reason, it was decided that the entire SFS structure should be raised 1.6 m above ground level to ensure floodwaters would not breach the structure even during heavy precipitation. The SFS was thus mounted on top of a 22 × 30 m steel-reinforced concrete platform of 0.16 m thickness. This platform was supported by 56 steel-reinforced concrete posts (1.1 m × 1.1 m) equidistantly spaced along the length and width which would allow for natural water flow to continue unimpeded under the structure during times of heavy floods.

**Figure 1:**
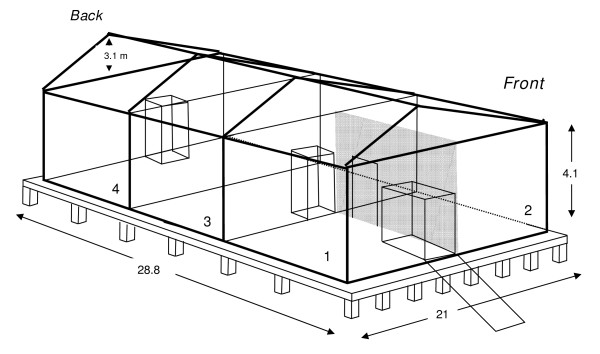
**Schematic diagram of the IHI Semi-field system (SFS) for research on African *Anopheles *ecology and control**.

The SFS outer was built from a pre-fabricated greenhouse frame (Shelter 9600, Filclair, Venelles, France). This structure originally consisted of 3 connected compartments of 9.6 × 21 m, but was modified by subdividing the first section into two units of 9.6 × 9 m and 9.6 × 12 m respectively (Figure 1). Rather than leaving the roof exposed to natural climatic conditions, it was covered with thick opaque white polyethylene plastic to guarantee protection from intense seasonal rains. The walls of the SFS were covered by PVC coated polyester netting of 346 holes per inch^2 ^(Polytex UK), which generates a mesh width approximately two times smaller than the standard recommended for bed nets [156 holes per inch2, [59]]. This product was selected on the basis that its filaments were woven together which prevents the mesh being stretched, its high degree of porosity (81%) which facilitates air movement, a shade factor of 56.5% to help reduce temperatures, and its UV-stabilization. After installation of the netting, data loggers (Tinytag TV-1500, Gemini Data Logger, UK) were placed in several areas of the SFS and surrounding outside environment to record temperature variation (taking readings approximately once every 10 minutes).

### Ethical considerations and community awareness

A potential risk of using SFS in disease-endemic settings is the accidental release of vectors into an environment where they could become infected with a human pathogen, increase the size of the local vector population or introduce a novel phenotype with enhanced transmission capacity. Consequently great care and vigilance is required to ensure the physical integrity of the structure and containment protocols. Access to the IHI SFS is restricted to a small number of research personnel. Research technicians conduct weekly intensive inspections of all areas of the inner and outer structure for physical damage that could allow mosquitoes to escape or enter from outside.

In addition to making sure that mosquitoes do not escape from a SFS, it is also imperative to ensure that malaria parasites are not accidentally introduced through mosquito contact with an infected person. A protocol for weekly malaria screening for all those working within the SFS was developed. Individuals found to be infected during this screening would be immediately treated with appropriate first line anti-malarial medication and excluded from the screen-houses for one month. Should it be found that a staff member has had malaria parasites while working within the SFS, the experimental chamber in which they worked can be shut down and all mosquitoes within it killed (by depriving them of water, blood and breeding sites for at least two weeks) to ensure no potentially infectious mosquitoes remain within it. Additional methods to reduce the risk of the unintentional introduction of parasites include the use of non-amplifying animal hosts such as livestock as the main blood source for captive vector populations. This procedure is being adopted in the IHI SFS where cows are used as the blood source for free-living *Anopheles *populations.

In addition to the precautions described above, a key ethical requirement of working with SFS is the creation and maintenance of strong support and awareness within the local community for these research activities. A series of public meetings with IHI staff, workers involved with the construction of the SFS, district health and government officials, and local residents were held in which information on the function and purpose of the SFS was disseminated. Ethical clearance from both the IHI Institutional Review Board (IHDRC/EC4/CL.N96/2004) and Tanzanian National Institute of Medical Research (NIMR/HQ/R.8a/Vol.IX/345) for SFS studies was obtained before the start of this study.

## Results

### Constructing the SFS

**Figure 2:**
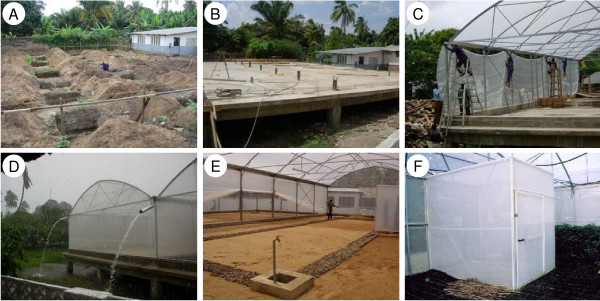
**Key steps in the construction of the IHI SFS.** (a) digging holes for foundation posts, (b) pouring the concrete foundation platform, (c) installing the netting, (d) roof gutters draining precipitation during peak rainfall, (e) French drain system installed under soil to divert surface water run off, (f) double entry door system.

**Figure 3:**
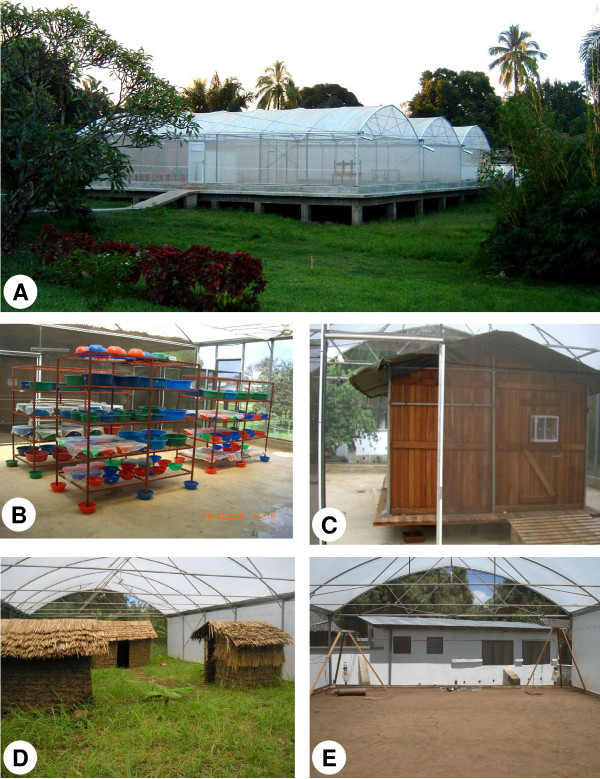
IHI SFS on completion (a) outer structure, (b) insectary section with thatched roof, (c) experimental hut trial area, (d) section for establishment of free-living, self-replicating *An. arabiensis *population, (e) section for olfaction and chemical ecology research.

Construction of the SFS began in July 2005. Work began by clearing all vegetation from the site, leveling the ground, and digging 56 holes (1 m depth) in the soil for the foundation platform posts (Figure 2a). Due to limited access to cement mixers and a constant supply of electricity, all cement required for the construction (approximately 250 m^3^) was mixed and poured by hand (Figure 2b). Approximately 20 full-time labourers were engaged in constructing the foundation over a 3-month period. Once the foundation had been completed, the pre-fabricated greenhouse frame with netting fitted was assembled over a period of 2 weeks (Figure 2c). Angled gutters were installed along the outside edge of each compartment to prevent the accumulation of rainwater on the roof (Figure 2d). Two electricity points were fitted into each compartment. Drainage and water pipes were fitted into each of the 4 compartments. Soil to a depth of 30 cm was added to sections 3 and 4 of the structure (Figure 1). Prior to adding soil to these compartments, sand and rocks were used to construct a drainage system to draw runoff from the soil towards outflow pipes (Figure 2e). Two main entrances were built at either end of the SFS, the front being accessible by a 6 m concrete ramp that permits livestock movement, and the posterior by stairs. Double-entry doors were constructed at both main entrances, and between section 1 and 3 (Figure 2f). The entire outer structure, including electricity and mains water supply was completed by October of 2006 (Figure 3a).

### Establishing research activities

Different research activities were allocated to each of the four SFS sections on the basis of maximizing logistical efficiency and minimizing the risk of mosquito escape or entry from outside. The first section behind the main front access point (section 1, Figure 3b) was designated for use as an *An. arabiensis *insectary. All mosquitoes in this section are thus additionally contained either in adult cages, or larval trays covered with netting. Initial consultation with the greenhouse manufacturer suggested that the netted outer walls would allow temperatures inside the SFS to equilibrate with ambient conditions outside. Ambient temperatures during the hot rainy season in Ifakara can exceed 40°C for several hours each day which are sufficiently high to kill adult and larval mosquitoes. To buffer the insectary from extreme temperatures that could knock out the colony, a traditional thatch roof was built within the insectary area to provide additional shading and cooling (Figure 3b). Under these conditions, an average of 638 pupae per day (± 114.8) were obtained from the F1 generation of *An. arabiensis *collected from a nearby village in March 2008.

The insectary connects directly onto two experimental spaces; the first being a 9.6 m × 10 m chamber within which an experimental hut (3.5 m × 4 × 2.5 m) was constructed for studies of mosquito host seeking and house entry behaviour (Figure 3c). This hut was fitted with 6 window traps that can be used to capture mosquitoes leaving and/or attempting to enter the house while a live host is within it [60]. This experimental hut section is designated for short-term behavioural studies in which no more than 300 mosquitoes at a time are released (at dusk), and subsequently recaptured the next day and removed in a cage. A further screen door separates this section from the insectary area meaning that three security doors must be passed through before reaching the outside, and minimizing the risk of mosquito escape during exit or entry.

The insectary also connects directly to a 9.1 × 21 m chamber designated for establishment of a free-living, self-replicating *An. arabiensis *population within a realistic ecosystem (Figure 3d). This section is intended for study of *Anopheles *behaviour, ecology and gene flow within an environment that mimics the natural surroundings as closely as possible. The exact number of free-living mosquitoes that will be held within this unit is uncertain, and will depend upon the carrying capacity of the established population at equilibrium. This section is linked to the main insectary by another double entry door system, requiring four doors to be passed through before reaching outside.

The fourth experimental section (9.1 × 21 m) is set up as an stand-alone experimental unit isolated from all other areas of the SFS, within which studies of olfaction and chemical ecology are ongoing (Figure 3e). This section is physically separated from the adjoining central section by thick polyethylene plastic which minimizes the direct flow of air and odours between them. Entry into this section is possible only from the rear SFS double entry door, and not through any other adjoining section. Studies using odour-baited traps to compare the attraction and repellency of different compounds to *Anopheles gambiae *s.s. are being conducted in this section.

### Replicating the natural environment

**Figure 4:**
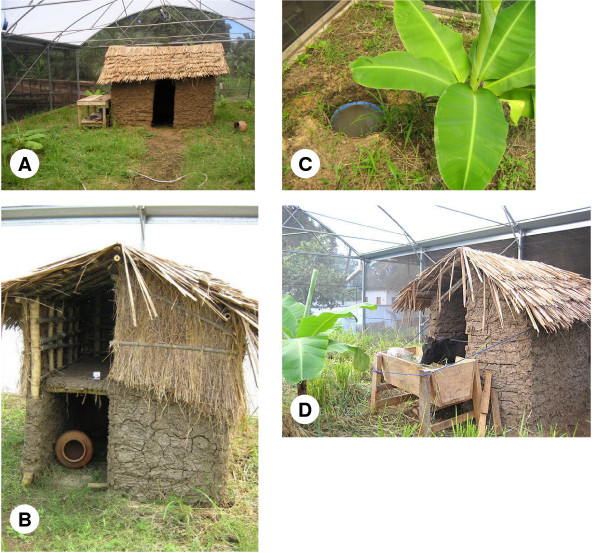
Habitat features within the SFS section designated for a free-living *An. arabiensis *population: (a) traditional mud-walled house, (b) chicken coop with clay pot refugia, (c) artificial breeding site and banana plant, (d) Cattle shed containing calf host.

As described above, one section of the SFS was set aside for establishment of a free-living population of *An. arabiensis *within conditions that mimic those of the natural environment. To achieve this, a domestic compound consisting of a mud-walled, thatched-roof house (2.6 m × 3 m × 2.5 m, Figure 4a), a typical outdoor toilet (1.4 m × 1.7 m × 2 m), and traditional chicken coop (1.8 m × 1.9 m × 2 m, Figure 4b) were constructed within this section by local builders. Grasses and other plants that emerged from the soil brought in from the local environment were allowed to grow. Additional plants common to the surrounding environment such as banana (Figure 4c), potatoes, rice, and castor bean (*Ricinus communis *L.) were introduced. A sprinkler system was installed so that varying levels of rainfall could be simulated. Five breeding sites were created by burying plastic buckets into the soil (50 cm diameter), adding 5 cm of soil, and filling them with water to a depth of 25 cm (Figure 4c). As *An. arabiensis *is somewhat zoophilic [61-64], regular blood meals can be provided to free-living mosquitoes within this section by introducing a cow or calf for a few nights each week (Figure 4d).

### Climatic conditions

**Figure 5:**
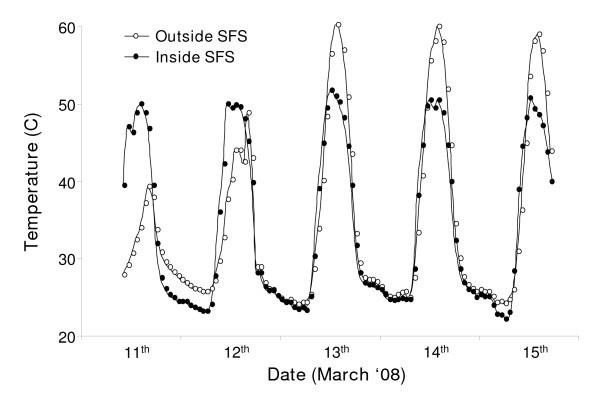
Average hourly temperatures at ground-level within the central section of the SFS and a nearby site outside of the SFS (3 m away) from May 9 – 14^th ^2008.

**Figure 6:**
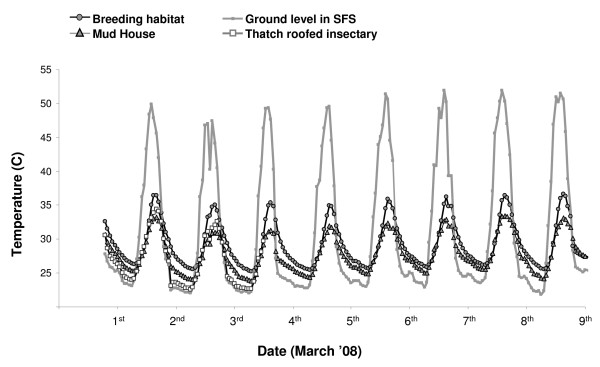
Average hourly temperatures at different locations within the SFS in the period from Feb 29^th ^– March 9^th ^2008.

A primary aim was to create climatic conditions within the SFS representative of the natural environment within the Kilombero region. Initial consultations with the greenhouse manufacturers indicated that the netting walls would allow temperatures inside the SFS to equilibrate with those outside. However, hot temperatures substantially higher than what is generally deemed acceptable for *An. gambiae *and *An. arabiensis *survival and reproduction (e.g. > 30°C for several hours each day) were soon observed within the SFS. These periods of high temperature, however, were similar to those of ambient conditions nearby but outside of the SFS where temperatures at ground level exceed 40°C up to 8 hours each day during the hot rainy season (Figure 5). Thus, although temperatures within the SFS were above the threshold for adult mosquito survival for periods of the day, they did not in general differ in mean or variability from those experienced in the nearby environment (e.g May 9–14^th ^2008: mean temperature inside SFS: 34.24°C ± 10.64°C SD, mean temperature outside the SFS: 34.33°C ± 11.20 SD). For mosquitoes to survive periods of excessively high temperatures both in nature and within the SFS, environmental refugia of substantially lower and less variable temperatures such as houses must be available [65]. The simple shaded refugia that were constructed within several areas of the SFS successfully reduced temperatures to within the acceptable range for adult and larval survival (Figure 6). The average temperature within the mud-walled house in the central SFS section was 3.5°C lower than within exposed areas of the SFS, and was substantially less variable (Table 2). Notably, temperatures inside the mud house did not exceed 35°C which is a critical threshold above which *An. arabiensis *in the laboratory begin to exhibit avoidance behaviour [66]. At 29.20°C (± 3.29°C), the average temperature in our artificial larval habitat was also within the natural range observed in *An. gambiae s.l. *aquatic habitats in east Africa, and did not exceed the upper tolerable limit of 40°C [67] (Table 2, Figure 6). The construction of a simple thatched roof over the insectary section of the SFS reduced temperatures by approximately 4°C in comparison to exposed areas of the SFS (Table 2, Figure 6), and considerably reduced the maximum temperature from 51.91°C to 34.69°C. Thus the climatic conditions within the SFS successfully represented the range of temperature extremes experienced in nearby field conditions, while providing realistic environmental refugia with temperatures appropriate for mosquito growth, survival and reproduction.

**Table 2:** Average temperatures at different locations within the SFS from February 29^th ^– May 9^th ^2008.

Location	Average Temperature (°C)	Standard Deviation (°C)	Range (°C)
Ground-level in SFS	31.24	9.62	21.77–51.91
Inside mud house	27.84	2.66	23.86 – 34.69
Artificial breeding site	29.20	3.29	25.19 – 36.67
Thatched-roof insectary	26.72	3.58	22.60 – 34.43

## Discussion

In just under two years a 625 m^2 ^state-of-the-art SFS for large-scale experimentation on anopheline mosquito ecology and control was established within a remote area of southern Tanzania where malaria transmission intensities are amongst the highest ever recorded [49-52,68]. This unique facility is more than 4 times larger than any SFS previously or currently in existence, and has capacity for a wide variety of research activities including mass-rearing of African malaria vectors under natural conditions, high throughput evaluation of novel control and trapping techniques, short-term assays of host-seeking behaviour and olfaction, and long-term experimental investigation of anopheline population dynamics and gene flow within a contained environment that simulates a local village domestic compound. This was accomplished through a multidisciplinary collaboration between entomologists, senior public health scientists of the IHI, architects, engineers, site managers and a dedicated team of labourers who built this structure largely in the absence of electricity or any other mechanized construction aids.

Experimental activities have only recently been initiated within the SFS, and the ultimate value of this facility as a research tool will be realized as the studies now underway reach conclusion. Preliminary results from short-term behavioural assays of *An. gambiae *host-seeking behaviour using odour-baited traps and live animal baits suggest that realistic and repeatable results can be obtained within the SFS in a relatively short period of time (I. Lyimo & S. Moore, pers. comm.). The longer-term task of establishing a self-replicating, free-living population of *An. arabiensis *within simulated village conditions is currently underway. Although too early to forecast the outcome of this objective, the fact that an amenable spectrum of climatic conditions can be generated within the SFS is encouraging. Although daily temperatures within exposed areas of the SFS routinely exceeded the optimum temperature of *An. gambiae *[26.5 C under insectary conditions, [69]] for some periods of the day, they were not significantly higher than those of the natural environment immediately outside of the SFS. Had the aim been to create an insectary facility for efficient mass production of *An. gambiae*, the regular daily periods of excessive ambient temperatures within the SFS (>35°C) would be a cause for concern. However the goal was instead to simulate the ambient climatic conditions within the Kilombero region and this was accomplished. Furthermore, the features that were built within the SFS provided microclimatic refuges in which mean temperature and variability was substantially reduced and stayed within the acceptable limits for adult survival and reproduction. For example, the mean temperature within the mud house inside the SFS was 3.5°C lower than exposed areas of the SFS. This observation matches reports from South Africa of air temperature inside mud and thatch houses being 3–6°C cooler than ambient conditions [70]. Temperatures within the mud house in our SFS were significantly higher than those reported in a similar structure within the SFS at Mbita, western Kenya [33], which may be more reflective of the different climatic conditions between study sites than structural differences in SFS design. Water temperatures within the artificial larval habitats in our SFS were higher than the reported optimal value gauged from insectary studies [24–26 C, [71]] and those reported for the Mbita SFS [33], but remained within the natural range observed in *An. gambiae s.l. *larval habitats in east Africa [72,73]. Given that free-living *An. gambiae s.s. *were able to complete their life cycle within the slightly cooler and much smaller confines of the Mbita SFS [33] there is optimism that the same can be achieved in the IHI SFS with *An. arabiensis*, a species known to have greater tolerance of hot and arid environments [66,74].

While early observations are promising, much still remains to be known about how representative conditions inside the SFS will be of mosquito ecology in the wild. Open questions include whether a self-replicating population can be maintained over numerous generations on this spatial scale, what carrying capacity this population will reach under ambient climatic and host (bovine) conditions, whether additional climatic refugia or controls will be needed, and if existing plant and nectar sources within the SFS will be sufficient to maintain the adult male population. Importantly, the identification of limitations in the ability of our SFS to replicate natural mosquito dynamics as experimental work progresses will in itself provide valuable knowledge of the crucial determinants of anopheline population growth and stability that would not be possible under natural field conditions.

As research begins at the SFS in Ifakara and similar facilities around the world (Table 1), it is useful to consider the major challenges to the successful use of this research tool. These challenges are varied and range from the purely scientific to those of logistics and ethics. Five key areas merit discussion. The first is the possibility that although biological inferences made from SFS may be much more realistic than those from cage studies, they may still misrepresent some areas of mosquito ecology and population processes in nature. For example, although full life cycle completion of *An. gambiae s.s. *was achieved within the SFS in western Kenya, it was noted that the artificial breeding sites within it gave rise to considerably fewer larvae than expected [33]. Whether this reduced efficiency was due to a problem with the environmental conditions inside the SFS, or maladaptation of the laboratory population used in these experiments to ambient conditions is unknown, but suggests there could be unique constraints or bottlenecks acting on population growth within these systems. Conversely, absence of the full range of environmental risks within the SFS such as stochasticity in host encounter rates, insecticide treated bed nets, predation by small vertebrates, pathogens and extreme environmental conditions such as flooding may result in an overestimation of life-history and demographic rates. For example, Knols *et al *estimated the daily survival of *An. gambiae s.s *within their SFS to be 90% which is higher than reported in many field studies [75]. In order to reduce the risk of accidental parasite introduction, non-amplifying animal hosts will be used as the main source of blood in many SFS. While numerous disease vectors include the blood of non-human animals in their diet, many of the species that are most problematic exhibit a pronounced preference for humans [76]. Several studies have shown that the fitness haematophagous insects derive from blood varies with host species [77-82]. It is also known that selection for divergent preference for human or cow hosts in *An. gambiae *mosquitoes can be generated in as little as 5–6 generations of selection [83]. Thus constantly exposing vector populations within SFS to non-human hosts could result in the generation of individuals with different phenotypes, genotypes and population dynamics than those who feed on and transmit disease to humans. Continued monitoring and comparison of SFS results to those observed in the field will be useful to identify which, if any, of these issues pose serious obstacles to interpretation and reinforce the point that SFS studies are intended to complement but not replace field studies.

A second scientific concern is that vector populations established within SFS will likely be considerably smaller than those in the wild and thus experience inbreeding and a resultant reduction in genetic diversity which could impede fitness. It is well known that genetic diversity within insect vectors can be considerably reduced during laboratory colonization [84-86]. Free-living populations established within SFS may be considerably larger than typical laboratory colonies and thus avoid a similar intensity of inbreeding, however it is unlikely they will escape some bottle-necking and an associated loss of diversity from founder populations. Within only a few generations of laboratory colonization, mosquitoes can develop significant behavioural divergence from wild populations which restricts mating between them [87]. This phenomenon may also occur within SFS, although perhaps at a slower rate than in small laboratory cages. As genetic and phenotypic divergence between contained SFS and wild populations may be unavoidable, the need for repeated comparative sampling of individuals in both settings is advocated to track if and how genetic diversity is reduced in captivity, and provide guidelines for how frequently captive populations should be enriched by fresh genetic material to maintain representative levels of diversity.

Should self-replicating vectors be successfully established in SFS, a logistical obstacle to the estimation of precise demographic rates from them will be the problem of disentangling overlapping generations. While much more precise estimates of mosquito population size will be possible within the contained environment of an SFS than in nature, it will remain difficult to accurately monitor individual-level activities such as mating behaviour and resource acquisition, and its resultant impact on fitness. The development of novel marking schemes using stable isotopes [88,89] or distinct genetic traits may permit more precise monitoring of the behaviour and reproductive success of specific subsets of individuals, or individuals themselves.

For greatest public health relevance, SFS should be situated within or as near as possible to natural disease transmission environments as possible. Placing a large contained population of competent disease vectors within an appropriate transmission setting will always raise biosecurity concerns. Any breach of containment could result in increasing the disease transmission within the local area, and the accidental introduction of parasites into contained populations from asymptomatic carriers working within the facility could also generate the potential for infection. Awareness and discussion of how to prevent those risks are absent from early accounts of SFS use, but are justifiably coming to the forefront as plans for large-scale studies with genetically-modified disease vectors come under development. Recently an international committee of scientists formalized guidelines on recommended biosecurity measures and precautions for contained SFS trials with genetically-modified mosquitoes [55]. The publication of these guidelines represents a significant step forward in thinking regarding the ethical responsibility for good practice within these facilities.

A final, crucial issue for the expansion of SFS-based research programmes throughout the world is the need to engage and promote awareness within the communities that host these facilities. The communities surrounding SFS research facilities should be the primary beneficiaries of research conducted within them, and their particular needs as end-users must be kept in mind when using these facilities to trial new vector control strategies. While researchers working in SFS may have this goal clearly in mind, it will be of little value unless clearly and regularly communicated to local communities in an open and discursive manner. Understandably local residents may be apprehensive about the placement of an SFS containing live insect vectors near their home, and misinformation about the purpose of this work and risks associated with it could cause considerable friction. This lesson was painfully learnt by scientists working at the Indian Council for Medical Research in the 1970's who had their research unit on *Aedes *mosquitoes shut down when journalists falsely alledged that the actual purpose of the work was biological warfare and human population control [41]. That such a debacle could occur in one of the most successful disease vector control research programmes of all time [with 104 papers published in 6 years and numerous insights into population suppression gained, [41]] is a sobering thought for all those involved in this new generation of SFS research. Community awareness activities have begun in Ifakara, and must be sustained and scaled up if both local community members and researchers working at the SFS are to obtain maximum benefits from this research facility.

Engagement must extend beyond local communities to include scientists and students working within the disease endemic countries that host SFS. These facilities can provide substantial indirect benefits by acting as state-of-the-art training tools for young vector biologists in which methodological skills can be honed, and independent research hypotheses experimentally tested in a disease-free setting. Currently, at the IHI, there are three east African postgraduate students pursuing their PhD studies on research based within the SFS and plans to recruit several more underway. Thus this research tool will contribute to the IHI's goal of substantially increasing Ph.D-level capacity in malaria vector research within Tanzania and east Africa. Much of the recent motivation for initiating SFS programmes has been driven by laboratory-based research on genetic modification of disease vectors that has occurred almost exclusively in developing countries. For both the transgenic approach and other emerging vector control strategies to fulfill their potential, it is absolutely imperative that endemic country scientists are actively involved in driving SFS-based research and taking forward innovative techniques developed within it.

## Competing interests

The authors declare that they have no competing interests.

## Authors' contributions

HMF was the primary project coordinator of SFS establishment at the IHI and drafted the manuscript. KRN designed and set up the areas of the SFS where a free-living *An. arabiensis *population will be established in simulated village conditions, and assisted with collection of temperature data. TW was the lead architect and DK the head of the maintenance unit that carried out construction. SJM oversaw parts of the construction and set up the olfaction study chamber. IL designed and set up the experimental hut and insectary area of the SFS. TR oversaw parts of the construction and helped supervise research activities within it. HU and HM provided institutional support and guidance in logistics, ethics, and community sensitization. GFK provided support with project planning and coordination. BGJ initiated this project, obtained financial support for it, and provided scientific and logistical guidance.
